# Assessing Engagement in Chinese Upper Secondary School Students Using the Chinese Version of the Schoolwork Engagement Inventory: Energy, Dedication, and Absorption (CEDA)

**DOI:** 10.3389/fpsyg.2021.638189

**Published:** 2021-02-18

**Authors:** Ziwen Teuber, Xin Tang, Katariina Salmela-Aro, Elke Wild

**Affiliations:** ^1^Department of Psychology, Bielefeld University, Bielefeld, Germany; ^2^Faculty of Educational Sciences, University of Helsinki, Helsinki, Finland; ^3^School of Psychology, Central China Normal University, Wuhan, China

**Keywords:** Chinese adolescents, well-being, high school students, vocational school students, depression, school engagement, academic engagement

## Abstract

The schoolwork engagement inventory: Energy, Dedication, and Absorption (EDA) is a measure of students' engagement in schoolwork and has been demonstrated valid in Western student populations. In this study, we adapted this inventory to and tested its psychometric appropriates in Chinese upper secondary school students (CEDA). Participants were 1,527 general high school students and 850 vocational high school students. The mean age of the total sample was 16.21 years (54.4% females, age span: 15–19 years). The results of confirmatory factor analyses (CFA) showed that a modified one-factor model fitted the data best. The results of the multigroup CFA showed that the factor structure was metrically invariant across school tracks (i.e., general or vocational high school) and scalarly invariant across gender and school types (i.e., ordinary or key school). Moreover, schoolwork engagement was negatively related to emotional exhaustion and positively related to self-efficacy, perseverance of effort, teacher–student relationships, and life satisfaction. Overall, the CEDA can be regarded as a valid measure for the assessment of student engagement in the Chinese upper secondary school context.

## Introduction

Being actively engaged in school and learning is vital to students' educational success and positive development (Bask and Salmela-Aro, 2013; Salmela-Aro and Upadyaya, 2014). Students' engagement with school has been seen as an antipode of academic and psychological maladjustment such as school burnout (e.g., Bask and Salmela-Aro, 2013) and an “antidote” of school dropout (Fredricks et al., 2004, p. 60). Over the last decades, it has been examined with great interest by Eastern and Western researchers (Fredricks et al., 2004; Zhang et al., 2007; Tuominen-Soini and Salmela-Aro, 2014; Cadime et al., 2016; Engels et al., 2016). However, with respect to the measurement of engagement, most measures originate in Western research communities. To date, although Chinese scholars have translated several commonly used measures into Chinese, most validation studies have focused on middle school students or general high school students (e.g., Wang and Eccles, 2012; Li et al., 2017). In this study, we introduce a Chinese version of the schoolwork engagement inventory: Energy, Dedication, and Absorption (CEDA) and comprehensively validated it by (1) examining the factor structure of the CEDA; (2) testing the measurement invariance of the CEDA across school tracks (i.e., academic vs. vocational tracks), school types (i.e., ordinary vs. key schools) and gender; and (3) testing the construct validity of the CEDA through exploring the relationship between student engagement and assumed correlates.

### Engagement and Assessment of Engagement

Engagement, the state of being involved, engrossed, and committed, has received increasing examinations in the past two decades in many research fields (e.g., educational and occupational contexts; Fredricks et al., 2004; Schaufeli, 2013; Wang et al., 2019). In the literature, school engagement is typically defined as a multidimensional construct that consists of academic (e.g., homework completion and time on task), affective (identification with school and school connectedness), cognitive (e.g., self-regulation and value of learning), and behavioral (e.g., attendance and extracurricular participation) components (Jimerson et al., 2003; Fredricks et al., 2004; Appleton et al., 2006). Although school engagement has been conceptualized as multidimensional, scholars have recognized that these subtypes are overlapping and interact with each other (Fredricks et al., 2004; Skinner and Pitzer, 2012).

Salmela-Aro and colleagues (Salmela-Aro et al., 2009; Salmela-Aro and Upadyaya, 2012; Tuominen-Soini and Salmela-Aro, 2014; Salmela-Aro and Vuori, 2015) proposed a concept of schoolwork engagement comprising energy, dedication, and absorption in schoolwork. This conceptualization stemmed from the research of work engagement which was defined as “a positive, fulfilling work-related state of mind that is characterized by vigor, dedication, and absorption” in the occupational context (Schaufeli et al., 2006, p. 702). In the concept of schoolwork engagement, energy (affective component) is characterized by high levels of vigor and mental resilience in the learning process. Dedication (cognitive component) refers to a positive cognitive attitude toward learning (e.g., perceiving schoolwork as meaningful, significant, and inspiriting). Absorption (behavioral component) is characterized by high concentration on learning, whereby time passes quickly.

In the present study, a focus is put on the Schoolwork Engagement Inventory: Energy, Dedication, and Absorption from Finland (EDA; Salmela-Aro and Upadyaya, 2012), which was constructed on the basis of the Utrecht Work Engagement Scale from the Netherlands (UWES; Schaufeli and Bakker, 2004). The original UWES contains 17 items and has been translated into 11 languages and validated in different cultures (Schaufeli and Bakker, 2004). The EDA contains nine items by selecting the most characteristic items of each dimension from the UWES in the school context. Recently, the EDA has been translated into German (Teuber et al., 2020a). Hence, the EDA enables an economic, valid, and comprehensive assessment of schoolwork engagement as well as cross-cultural comparisons.

The multidimensional but overlapping dimensions are also reflected in the measurement of work engagement and schoolwork engagement. In several large validation studies, Schaufeli and colleagues (Schaufeli et al., 2002a,b, 2006) tested the factorial structure of the UWES in employees and university students and suggested that the one-factor structure best fitted the data for younger students and gave the best reliability and validity indices. In the validation study of the Finnish EDA (Salmela-Aro and Upadyaya, 2012), the authors recommended a one-factor solution yielding excellent reliability and validity. The authors assume that this result is due to the fact that younger students cannot comprehend the subtleties in the engagement concept and suggest that the three dimensions still effectively describe student engagement. Hence, in many studies, the overall scale of the EDA has been used for the assessment of students' engagement (Salmela-Aro et al., 2009; Salmela-Aro and Vuori, 2015; Tang et al., 2019; Teuber et al., 2020a).

To date, it still lacks evidence about the dimensionality of schoolwork engagement in Chinese upper secondary school student population. Moreover, most engagement research in Chinese upper secondary schools has been mainly focused on students in the general high schools, not the vocational high schools [e.g., Wang and Eccles (2012) and Li et al. (2017)]. The present study aims to fill those research gaps by validating EDA in the Chinese high school contexts and by testing the engagement from both general high and vocational high schools.

### Correlates of Engagement

Apart from the assessment of student engagement, researchers are also interested in the antecedents, mechanism, and consequences of student engagement. Several models have been established to address these aspects [e.g., Skinner (2016) and Wang et al. (2019)]. One of the most popular heuristic models—-the Job Demands-Resources Model (Bakker and Demerouti, 2014)—provides a theoretical foundation to study correlates of engagement. We choose this model as it is the most common model to examine work engagement using the UWES—the foundation of EDA. In the heart of the Job Demands-Resources Model lies the assumption of two independent psychological processes: In the *health impairment process*, job demands require physical and psychological effort and can lead to strain, which in turn can cause negative psychological and occupational outcomes. In the *motivational process*, job and personal resources have a motivational role and lead to higher levels of engagement, which in turn has a positive effect on one's psychological and occupational outcomes. There is strong cross-sectional and longitudinal evidence supporting the assumed two psychological processes in a variety of job settings [for an overview, see Bakker and Demerouti (2014)].

Recently, the Job Demands-Resources Model has been successfully adapted to both Western (Tuominen-Soini and Salmela-Aro, 2014; Gusy et al., 2019; Lesener et al., 2020; Teuber et al., 2020a) and Asian (Teuber et al., 2020b) educational contexts, which was also renamed as School Demands-Resources Model (Salmela-Aro and Upadyaya, 2014). Using the School Demands-Resources Model as a framework model, several positive psychological traits have been identified as personal resources that promote student engagement. For instance, Salmela-Aro and Upadyaya (2014) found in their longitudinal study that Finnish upper secondary school students' self-efficacy predicted their engagement in schoolwork, and Teuber et al. (2020b) found a potential engagement-enhancing effect of students' perseverance of effort or perseverant grit (i.e., the tendency to work hard even in difficult situations; Duckworth et al., 2007). In addition to personal resources, there are also resources in the school environment. For instance, an affectively close and supportive teacher–student relationship (Roorda et al., 2011, 2017) and social support from peers (Gusy et al., 2016; Teuber et al., 2020a) have been found to contribute to students' engagement.

Together with school burnout (a symptom characterized by emotional exhaustion at schoolwork, cynicism toward the meaning of school, and sense of inadequacy at school; Salmela-Aro et al., 2009), students' engagement with schoolwork affects their academic outcomes. In a longitudinal study, Tuominen-Soini and Salmela-Aro (2014) investigated patterns of student engagement and school burnout among *N* = 979 participants (mean age = 18.11 years) in high school. Six years later, 68% of the original participants were involved in the second wave. Using latent profile analyses, four groups of students in high school were identified: engaged, engaged–exhausted, cynical, and burned-out (high scores on all three dimensions of burnout: emotional exhaustion, cynicism, and reduced personal accomplishment) students. The results demonstrated that both engaged students were successful in school, although engaged–exhausted students were more stressed, whereas cynical and burned-out students were less engaged and had lower academic achievement. Six years later, engaged students were more likely to attend university. Furthermore, results indicated that the patterns were rather stable over time from late adolescence to young adulthood.

Apart from the impact on academic outcomes, previous research has linked schoolwork engagement to various psychological outcomes. Within the School Demands-Resources framework, longitudinal (Salmela-Aro and Upadyaya, 2014) and cross-sectional studies (Teuber et al., 2020b; Tuovinen et al., 2020) show that upper secondary school students who report higher levels of schoolwork engagement are more satisfied with their life and show fewer depressive symptoms than their less-engaged peers.

Based on the School Demands-Resources framework and previous findings, we aimed to test the construct validity of the CEDA by exploring its relations to demonstrated antipode, antecedents, and outcomes: emotional exhaustion (a core symptom of burnout), self-efficacy, optimism, perseverance of effort, teacher–student relationships, satisfaction with life, and depressive symptoms.

### Gender Differences in Engagement

In addition to understanding the correlates of engagement, gender differences of engagement are also an important topic to be examined. A large body of research on students' academic outcomes has reported that girls are more successful than boys in school. In a variety of education systems (e.g., Germany, England, and USA), girls are overrepresented in higher or academic school tracks, whereas boys are overrepresented in lower or vocational school tracks and have a higher risk of dropping out of school [for an overview, see Kessels et al. (2014)]. The recent PISA (Programme for International Student Assessment; OECD, 2019) reported again that in all PISA-participating countries, girls outperformed boys in reading. This is, however, not due to the intellectual level or gender-specific inequity in educational systems but rather due to the gender-specific difference in students' engagement (Lei et al., 2018). The results of a meta-analysis (Lei et al., 2018) on students' engagement showed that under the condition of the same level of intellective ability, females are more likely to be successful in education because they engage more in schoolwork than their male peers. A large cross-national study (*N* = 3,420 7–9th grade students from 12 countries, including China; Lam et al., 2014) on gender differences in student engagement indicated that compared to girls, boys are less engaged and interested at school, tend to find schoolwork less meaningful, and are less willing to spend time on it and that engagement partially mediates the relationship between gender and academic performance. Hence, it is reasonable to assume that in the Chinese upper secondary school context, girls show a higher level of engagement than do boys.

### Upper Secondary Education in China

The current study took place in upper secondary schools in China. After the completion of lower secondary school (also known as junior high school), which marks the end of a 9-year compulsory education, Chinese students have the possibility to continue with upper secondary education, which takes three years [for further reading, see OECD (2016)]. Recently, around 95% lower secondary school graduates continue their study in upper secondary schools. Broadly, there are two school tracks of upper secondary education: academic track (general high schools) and vocational track (vocational high schools). Whether a student can enter a general high school or a vocational high school is determined by the performance of an academic aptitude test—*zhongkao* (upper secondary school entrance examination). According to the statistics provided by the National Bureau of Statistics of China (2020; for more statistics, visit http://www.stats.gov.cn/english/), the nationwide enrollment rate of high schools in 2019 was 58.3%.

Compared to general high schools that are linked to a more promising future (e.g., access to higher education, associated with better chances on the labor market), vocational high schools have been associated with low educational quality, and their students are often considered inferior to general high school students in terms of academic performance (Zhang et al., 2015). In the last 25 years, vocational high schools have been expanded, since policymakers recognized that high-qualified vocational high school graduates were of high importance in ensuring a skilled labor force in China [for an overview, see Yi et al. (2013)]. However, for historical, social, and cultural reasons, it is still a long way to improve the quality and the reputation of vocational secondary education in China.

Another feature of the upper secondary education in China is that schools are categorized into key and ordinary schools. In comparison to ordinary schools, key schools have a better academic reputation and are usually allocated more resources (You, 2007). For example, key schools employ more highly qualified teachers (usually with a master's degree or higher) and receive more funds from the state input for science equipment, international student exchange programs, and native-speaking teachers in foreign languages.

Relying on the School Demands-Resources Model, learning environment affects students' academic and psychological adjustment. Although general high schools in China are more challenging (higher levels of academic demands) than vocational high schools, they have more highly qualified professionals including school psychologists who can offer professional instructional, social, and emotional support. The supportive environment may enhance students' engagement. Similarly, compared to ordinary schools, key schools are more competitive but provide a more stimulating learning environment. Against this background, we assume that general high school students in China are more engaged in their academic work than vocational high school students and that key school students are more engaged than ordinary school students.

### The Present Study

As previously mentioned, although schoolwork engagement is considered as a three-dimensional concept (i.e., energy, dedication, absorption), large validation studies have shown that a one-factor structure is more suitable in young student populations. There are an increasing number of studies involving this scale to measure student engagement in China. However, there is no attempt to comprehensively test its factorial structure in both Chinese general high school students and vocational high school students. This study aims to fill this gap and test the psychometric properties in a Chinese upper secondary school student population. We conducted this study in the following steps:

In the first step, we evaluated the factorial structure of the CEDA by comparing a one-factor model with a three-factor CFA model. Based on previous findings, we hypothesize that

- H1: A one-factor model is better than a three-factor model.

In the second step, we tested the measurement invariance of the CEDA across gender, school tracks (i.e., general vs. vocational high schools), and school types (i.e., ordinary vs. key schools). We assume that

- H2: Schoolwork engagement is at least metrically invariant across gender, school tracks, and school types.

In the third step, the criterion validity of the CEDA was tested by exploring its relationship with teacher–student relationships, self-efficacy, life satisfaction, perseverance of effort, emotional exhaustion, and depressive symptoms. Based on previous findings and the School Demands-Resources assumptions, we expect that

- H3: Schoolwork engagement is positively related to teacher–student relationships, self-efficacy, life satisfaction, and perseverance of effort, whereas schoolwork engagement is negatively related to emotional exhaustion and depressive symptoms.

If there is evidence supporting the validity of the CEDA, it allows us to test gender, school track, and school type differences in schoolwork engagement in the next step. We hypothesize that

- H4: Girls are more engaged in schoolwork than boys.
- H5: General high school students are more engaged in schoolwork than vocational high school students.
- H6: Key school students are more engaged in schoolwork than ordinary school students.

## Methods

### Data Collection

As previously mentioned, Chinese secondary schools are highly segregated according to students' academic performance. For example, in the Chinese municipality of Shanghai, according to the level of academic achievement, general high schools are divided into Shanghai key high schools (most resources and highest academic demands), district key high schools, and ordinary high schools (lowest resources and lowest demands). Compared to general high schools, vocational high schools are less segregated, and students inside a vocational school are more heterogeneous.

To collect data, we selected and contacted five high schools in Shanghai from a list of schools to make sure one is a Shanghai key school, two are district key schools, and two are ordinary schools. We chose one private school and one public school to ensure the representation of the ordinary schools. All schools agreed to attend this study. After that, two classes of each grade level in the respective school were randomly selected. Within these classes, all students were invited to participate in this study. In addition, one vocational high school in Panjin, Liaoning, was selected and contacted. The school agreed to participate in the study, and all students in this school were invited to attend this study.

As a result, this study involved one private ordinary high school, one ordinary high school, one campus district key high school (where students have the opportunity to live on campus), one district key high school, and one Shanghai key high school in Shanghai and one ordinary vocational high school in Panjin, Liaoning province (in northern China and comprises both rural and urban areas) to represent the differences in the Chinese high school system.

Previous to the data collection (from February to May 2019), this study was proven by the ethics review committee of Bielefeld University and received its permission. We contacted school principals in person or by telephone and informed about the purpose of this study. The schools distributed a letter to the students and their parents or guardians explaining the nature and design of the study. All participants were informed that they were free to withdraw from participation at any time without consequences. The students responded anonymously and voluntarily to the survey. To be eligible, participants and their parents or guardians had to provide informed consent.

### Participants

Participants were *N* = 2,377 Chinese school students (54.4% females, mean age = 16.21 years, *SD* = 1.95). Among them, 1,527 were students (52.40% females; age span: 15–19 years) from 42 classes in five high schools in Shanghai. Their mean age was 16.38 years (*SD* = 1.04). Around 11% of them were from the private ordinary high school, 44% from the ordinary high school, 16% from the campus district key high school, 9% from the district key high school, and 20% from the Shanghai key high school. The participation rate was 80.79%.

The other 850 were students (56.71% females) from 44 classes in the ordinary vocational school in Panjin. Their mean age was 16.66 years (*SD* = 0.78, age span: 16–19 years). This school provided 13 majors in total. The major distribution of the subsample was 42.50% preschool education, 20.03% automotive mechatronics, 4.97% graphic design, computer networks, 9.03% arts and crafts, 7.83% computer application, 6.93% accountancy, 3.01% cosmetology, 2.26% tourism service and management, 1.96% food technology, and 1.48% else. The participation rate was 89%.

Table 1 provides more details about the samples. The socioeconomic status of the total sample was approximately normally distributed (skewness = −1.94, kurtosis = 0.21). All participants were native Mandarin speakers.

**Table 1:** Sample distribution.

	**General high school students**			**Vocational high school students**		
**Grade level**	**10th**	**11th**	**12th**	**10th**	**11th**	**12th**
Sample size	612	513	402	428	347	75
Number of girls	314	271	209	250	205	37
Mean age (*SD*)	15.54 (0.6)	16.43 (0.73)	17.58 (0.59)	15.48 (2.54)	16.65 (5.11)	17.77 (1.8)
Average SES (*SD*)	3.16 (1.04)	3.14 (1.12)	3.22 (1.11)	2.55 (1.25)	2.58 (1.27)	2.99 (1.24)

*SES, socioeconomic status. In this study, SES was operationalized as the number of books in households*.

### Measures

#### Schoolwork Engagement

To assess schoolwork engagement, we adopted the Schoolwork Engagement Scale (EDA; Salmela-Aro and Upadyaya, 2012). Because the EDA is based on the Utrecht Work Engagement Scale (UWES; Schaufeli and Bakker, 2004), we relied on the Chinese wording of the UWES and the item selection of the EDA. The term “work/job” was replaced by “schoolwork.” The concept of schoolwork engagement consisted of three dimensions with three items each: energy, dedication, and absorption. The Chinese items are shown in Table 2. Students responded on a 7-point scale (0 = *never*, 6 = *always*).

**Table 2:** Items of the schoolwork engagement inventory: energy, dedication, and absorption (EDA).

	**CEDA**	**EDA**
ENE1	在学校我感到自己迸发出能量	At school I am bursting with energy
DED1	我觉得我的学习目标明确, 且很有意义	I find the schoolwork full of meaning and purpose
ABS1	当我学习时, 时间总是过得飞快	Time flies when I am studying
ENE2	学习时, 我感到自己强大并且充满活力	I feel strong and vigorous when I am studying
DED2	我对学习富有热情	I am enthusiastic about my studies
ABS2	当我学习时, 我忘记了周围的一切事情	When I am working at school, I forget everything else around me
DED3	学习激发了我的灵感/求知欲	My schoolwork inspires me
ENE3	早上一起床, 我就想要去学习	I feel like going to school when I get up in the morning
ABS3	当学习紧张的时候, 我会感到快乐	I feel happy when I am working intensively at school

*ENE, energy; DED, dedication; ABS, absorption. The English items of the EDA are provided by Salmela-Aro and Upadyaya (2012)*.

#### Emotional Exhaustion

Emotional exhaustion was measured with the emotional exhaustion subscale of a Chinese version (Wu et al., 2010) of the Maslach Burnout Inventory-Student Survey (Maslach et al., 2006). It consisted of three 5-point items (e.g., “I feel emotionally drained by learning”). Its psychometric properties have been confirmed in previous studies (Teuber et al., 2020b). In the present analyses, a one-factor structure fitted the data well [χ^2^= 0.901, *df* = 1, *p* = 0.343, CFI = 1.000, SRMR = 0.016, RMSEA = 0.000, 90% CI for RMSEA (0.000, 0.053)]. McDonald's ω was 0.87.

#### Self-Efficacy

Self-efficacy was measured with the corresponding subscales of the validated Chinese Questionnaire to Assess Resources for Children and Adolescents (QARCA-C; Teuber et al., 2020c). This scale consists of six 4-point items (1 = *never*, 4 = *always*). An example item is “I can reach a lot with my ability.” Self-efficacy was unidimensional in this study [χ^2^ = 34.659, *df* = 6, *p* < 0.001, CFI = 0.993, SRMR = 0.013, RMSEA = 0.045, 90% CI for RMSEA (0.031, 0.06)]. McDonald's ω was 0.93.

#### Perseverance of Effort

To assess perseverance of effort, we used the validated Chinese version (Li et al., 2018) of the Short Grit Scale (Grit-S; Duckworth and Quinn, 2009). It comprised four 5-point items (1 = *not at all like me*, 5 = *very much like me*). An example item is “I finish whatever I begin.” A CFA indicated that a model with one latent factor fitted the data well [χ^2^ = 13.412, *df* = 2, *p* < 0.01, CFI = 0.992, SRMR = 0.014, RMSEA = 0.049, 90% CI for RMSEA (0.027, 0.076)]. McDonald's ω of the scale was 0.82.

#### Teacher–Student Relationships

Teacher–student relationships were measured with the corresponding 5-item scale used in the Chinese student questionnaire of the PISA study (OECD, 2012). This scale covered teachers' emotional and instrumental support, teachers' fairness, and the perceived quality of the teacher–student relationship in general (e.g., “If I need extra help, I will receive it from my teachers”). All items were coded on a 4-point scale (1 = *totally disagree*, 4 = *totally agree*). The result of CFA supported its unidimensional structure in the present study [χ^2^ = 40.319, *df* = 5, *p* < 0.001, CFI = 0.997, SRMR = 0.013, RMSEA = 0.055, 90% CI for RMSEA (0.040, 0.071)]. McDonald's ω of the overall scale was 0.95.

#### Life Satisfaction

Life satisfaction was assessed using the validated Chinese version (Pavot and Diener, 1993) of the Satisfaction with Life Scale (SWLS; Diener et al., 1985). The SWLS consisted of five items (e.g., “In most ways, my life is close to my ideal.” 1 = *strongly disagree*, 7 = *strongly agree*). In this study, this scale showed a unidimensional factor structure [χ^2^ = 66.894, *df* = 5, *p* < 0.001, CFI = 0.983, SRMR = 0.019, RMSEA = 0.073, 90% CI for RMSEA (0.058, 0.089)]. McDonald's ω was 0.94.

#### Depressive Symptoms

Depressive symptoms were examined with the Chinese version (Chen et al., 2009) of the Center for Epidemiologic Studies Depression Scale (CES-D; Radloff, 1977). The scale was designed for use in studies on the general (non-clinical) population. Previous analyses support a four-factor structure (Depressed Affect, Positive Affect, Somatic Symptoms/Retarded Activity, and Interpersonal) that can be represented by 20 items in a 4-point scale (e.g., “During the past week, I felt sad.” 0 = *rarely or none of the time*, 3 = *most or almost all the time*). The sum score ranged from 0 to 60, and a score equal to or above 16 indicated a person at risk for clinical depression. In Chinese adolescents, the Chinese CES-D shows very good psychometric properties (Chen et al., 2009). The 4-factor structure could be confirmed in our study [χ^2^ = 1525.996, *df* = 166, *p* < 0.001, CFI = 0.962, SRMR = 0.029, RMSEA = 0.059, 90% CI for RMSEA (0.057, 0.061)]. In this study, the sum score was used (McDonald's ω = 0.97).

#### Demographics

Demographic variables included sex (0 = *male*, 1 = *female*), school track (0 = *vocational high school*, 1 = *general high school*), and school type (0 = *ordinary school*, 1 = *key school*). To assess socioeconomic status (SES), we asked for the number of books in the home using the same item as in the Chinese PISA study (1 = *less than 20 books*, 5 = *more than 200 books*; OECD, 2013). A huge number of studies [e.g., OECD (2013)] show that this item is strongly correlated with parents' income and educational level and can, thus, be seen as a powerful indicator of SES.

### Data Analysis

Data analyses were conducted in Mplus 8 Muthén and Muthén, 1998–2020). We tested the item distribution and multivariate outliers. All variables were approximately normally distributed. Skewness and Kurtosis were between −0.37 and 0.43. We used the TYPE = COMPLEX (CLUSTER = class) option to respect the clustered data structure. Missing value analysis indicated that, for all variables, data were missing under 0.7% of the cases. Little's test (LIT) showed that the data had a Missing Completely at Random (MCAR) mechanism. Moreover, we used the maximum likelihood robust (MLR) as the estimator to retain the full information, and it is robust to non-normality of observed variables.

To analyze the factorial structure of the CDEA, we performed confirmatory factor analyses (CFA) with three models: (1) a one-factor CFA model in which all items weighted on one single factor; (2) a three-factor model comprising energy, dedication, and absorption as three correlated but distinct factors; and (3) a second-order CFA model. The final model was used in further analyses.

Before testing the measurement invariance across school tracks, school types, and gender, the final model from the last step was used as the baseline model and explored separately for the subsamples (i.e., general high school students and vocational high school students; ordinary school students and key school students; males and females). If the model is appropriate in the subsamples, testing measurement invariance would be possible. To test its measurement invariance, multigroup CFA models were performed (van de Schoot et al., 2012). First, all parameters were freely estimated (M1; configural measurement invariance). Second, factor loadings were constrained across the subgroups (M2; metric measurement invariance). Third, not only factor loadings but also indictor intercepts were constrained (M3; scalar measurement invariance).

To evaluate the model fit, we relied on the recommendations by Hu and Bentler (1999) with a non-significant χ^2^-value, a Comparative Fit Index (CFI) ≥0.95, Root Means Square Error of Approximation (RMSEA) ≤0.05, and Standardized Root Mean Square Residual (SRMR) ≤0.05 indicating good model fit and CFI ≥0.90, RMSEA ≤0.08, and SRMR ≤0.08 indicating acceptable fit. For model comparisons, the Satorra–Bentler scale χ^2^ difference test was used (Satorra and Bentler, 2010). Due to the fact that the χ^2^ statistic is sensitive to sample size, we additionally relied on Chen's (2007) recommendation for measurement invariance (sample size >300): A decrease of ≥0.010 in CFI, an increase of ≥0.015 in RMSEA, and an increase of ≥0.030 in SRMR indicate non-invariance.

For the evaluation of the construct validity of the CEDA, we investigated the relationships between schoolwork engagement, gender, school track, school type, socioeconomic status, emotional exhaustion, teacher–student relationships, self-efficacy, perseverance of effort, life satisfaction, and depressive symptoms using structural equation modeling (SEM).

## Results

### Descriptives

Mean scores and standard deviations of all variables for the total sample and the six participating schools are presented in Table 3. Table 4 shows intercorrelations between all scales (mean scores) in the total sample.

**Table 3:** Means and standard deviations (in parentheses) for the total sample and the six schools, respectively.

**Sample**	**CEDA**	**EE**	**SEFF**	**PER**	**TSR**	**SWLS**	**DEP**
Total	3.09 (1.30)	2.86 (1.15)	2.63 (0.71)	3.14 (0.85)	2.99 (0.78)	4.43 (1.48)	24.96 (16.10)
1	3.05 (1.34)	3.04 (1.17)	2.57 (0.70)	3.18 (0.83)	3.00 (0.60)	4.36 (1.43)	19.05 (11.68)
2	3.04 (1.22)	3.00 (1.10)	2.58 (0.68)	3.12 (0.78)	3.08 (0.60)	4.27 (1.45)	18.17 (11.38)
3	3.00 (1.14)	3.34 (1.04)	2.50 (0.56)	3.03 (0.67)	3.08 (0.54)	4.02 (1.49)	17.72 (11.06)
4	2.94 (1.01)	2.92 (1.00)	2.53 (0.61)	3.01 (0.71)	3.02 (0.54)	4.42 (1.35)	20.11 (10.97)
5	3.22 (1.15)	2.82 (1.13)	2.70 (0.65)	3.15 (0.73)	3.14 (0.56)	4.54 (1.34)	14.58 (10.13)
6	3.16 (1.48)	3.01 (1.11)	2.59 (0.66)	3.11 (0.76)	3.08 (0.58)	4.31 (1.43)	17.64 (11.20)

*1, private ordinary high school; 2, ordinary high school; 3, campus district key high school; 4, district key high school; 5, Shanghai key high school; 6, vocational school; CEDA, student engagement; EE, emotional exhaustion; DEP, depressive symptoms; SWLS, satisfaction with life; TSR, teacher–student relationship; SEFF, self-efficacy; PER, perseverance of effort*.

**Table 4:** Intercorrelations between all scales in the total sample.

	**CEDA**	**EE**	**SEFF**	**PER**	**TSR**	**SWLS**
EE	−0.30^**^					
SEFF	0.47^**^	−0.20^**^				
PER	0.56^**^	−0.13^**^	0.57^**^			
TSR	0.32^**^	−0.11^**^	0.37^**^	0.30^**^		
SWLS	0.38^**^	−0.21^**^	0.56^**^	0.44^**^	0.41^**^	
DEP	−0.24^**^	0.14^**^	−0.01^ns^	−0.03^ns^	−0.14^**^	−0.06^*^

* *p < 0.05*.

** *p < 0.001. CEDA, student engagement; EE, emotional exhaustion; SEFF, self-efficacy; PER, perseverance of effort; TSR, teacher–student relationship; SWLS, satisfaction with life; DEP, depressive symptoms. ^ns^not significant*.

### Factorial Structure

**Figure 1:**
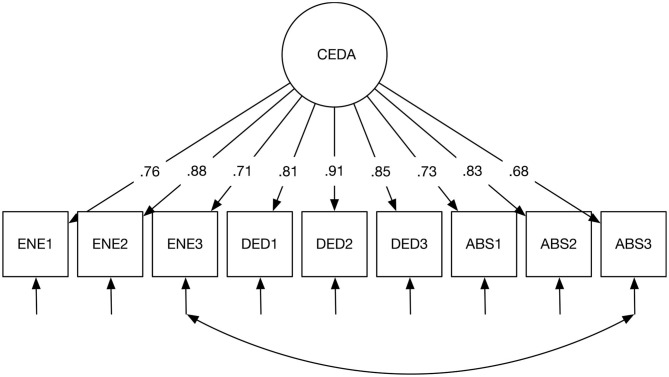
The final CFA model. All factor loadings were significant with *p* < 0.001.

As outlined above, we performed a one-factor CFA model, a three-factor CFA model, and a second-order CFA model to evaluate the factorial structure of the CEDA (see Table 5). The fit indices of all three CFA models were generally acceptable—with only RMSEA not meeting (but approaching) its criterion of 0.08. Moreover, the modification indices suggested that the fit of the models would improve significantly if the residual variance between the third item of energy (ENE3; “I feel like going to school when I get up in the morning”) and the third item of absorption (ABS3; “I feel happy when I am working intensely at school”) would be allowed. After we correlated those two items, the model fits improved drastically (see Table 5). Finally, we chose this modified one-factor model (see Figure 1) following the suggestion of Schaufeli et al. (2006). It is also important to note that in the three-factor model, the factors highly correlated with each other (standardized correlation over 0.95). Thus, the one-factor model was better to alleviate the possible multicollinearity problem than the three-factor model. All unstandardized parameter estimates of factor loadings in the modified one-factor model were statistically significant (*p* < 0.001). Standardized parameter estimates of scores of factor loadings ranged from 0.67 to 0.91. The modified one-factor model was retained for further analyses.

**Table 5:** Goodness-of-fit of the hypothesized and alternative models of CEDA in the total sample.

**Model**	**χ^2^/*df***	**CFI**	**RMSEA (90% CI)**	**SRMR**
One-factor model	558.681^*^/27	0.934	0.091 (0.085, 0.098)	0.043
Three-factor model	564.855^*^/24	0.933	0.097 (0.091, 0.105)	0.042
Second-order model	564.855^*^/24	0.933	0.097 (0.091, 0.105)	0.042
**Modified one-factor model**	257.578^*^/26	0.971	0.061 (0.055, 0.058)	0.030

* *p < 0.001. Bold font: final model*.

Cronbach's alpha for the overall student engagement scale was 0.95, and McDonald's Omega was also 0.95.

### Measurement Invariance

Following the analytical plan, we first estimated the final model from the last step as the baseline model separately high school students and vocational school students. The baseline model fitted the data well in both subgroups (see Table 6). The configural invariance held (M1). Compared to the unconstrained M1, the goodness of fit of M2 was close. Although Δχ^2^ was significant, the change of other fit indices was small (ΔCFI <0.010, ΔRMSEA <0.015, ΔSRMR <0.030). Hence, the fit of M2 to the data was as good as the fit of M1, indicating that the factor loadings were invariant across samples. In M3, all fit indices became worse meaning that the intercepts of the indicators were non-invariant across school tracks.

**Table 6:** Results of measurement invariance (MI) across school tracks.

**Model**	**χ^2^/*df***	**CFI**	**RMSEA (90% CI)**	**SRMR**	**χ^2^**
M0—high school	191.432^*^/26	0.965	0.065 (0.056, 0.073)	0.032	–
M0—vocational school	156.562^*^/26	0.969	0.077 (0.066, 0.089)	0.024	–
**Measurement invariance**					
M1: configural MI	345.114^*^/52	0.967	0.069 (0.062, 0.076)	0.029	–
M2: metric MI	391.986^*^/60	0.963	0.068 (0.062, 0.075)	0.039	46.872^*^
M3: scalar MI	528.728^*^/69	0.948	0.075 (0.069, 0.081)	0.053	136.742^*^

*M0, baseline model*.

* *p < 0.001*.

The baseline model fitted data of ordinary school students and key school students as well as girls and boys (see Tables 7, 8). The results of measurement invariance showed that a scalar measurement invariance held across gender and school types, indicating that the factor loadings and indicator intercepts were invariant across gender and school types.

**Table 7:** Results of measurement invariance (MI) across school types.

**Model**	**χ^2^/*df***	**CFI**	**RMSEA (90% CI)**	**SRMR**	**χ^2^**
M0—OS	202.044^**^/26	0.971	0.063 (0.055, 0.072)	0.027	–
M0—KS	96.833^**^/26	0.968	0.063 (0.050, 0.077)	0.032	–
**Measurement Invariance**					
M1: configural MI	311.243^**^/52	0.971	0.065 (0.058, 0.072)	0.029	—
M2: metric MI	334.356^**^/60	0.969	0.062 (0.056, 0.069)	0.037	23.113^*^
M3: scalar MI	383.385^**^/69	0.965	0.062 (0.056, 0.068)	0.042	49.029^*^

*M0, baseline model; OS, ordinary school; KS, key school*.

* *p < 0.01*,

** *p < 0.001*.

**Table 8:** Results of measurement invariance (MI) across gender.

**Model**	**χ^2^/*df***	**CFI**	**RMSEA (90% CI)**	**SRMR**	**χ^2^**
M0—males	121.757^**^/26	0.975	0.058 (0.048, 0.069)	0.025	–
M0—females	187.783^**^/26	0.966	0.070 (0.060, 0.079)	0.032	–
**Measurement Invariance**					
M1: configural MI	305.424^**^/52	0.970	0.064 (0.057, 0.071)	0.029	—
M2: metric MI	335.805^**^/60	0.968	0.062 (0.056, 0.069)	0.034	30.381^*^
M3: scalar MI	378.607^**^/69	0.964	0.062 (0.056, 0.068)	0.035	42.802^*^

*M0, baseline model*.

* *p < 0.01*,

** *p < 0.001*.

### Relations With External Criteria

**Figure 2:**
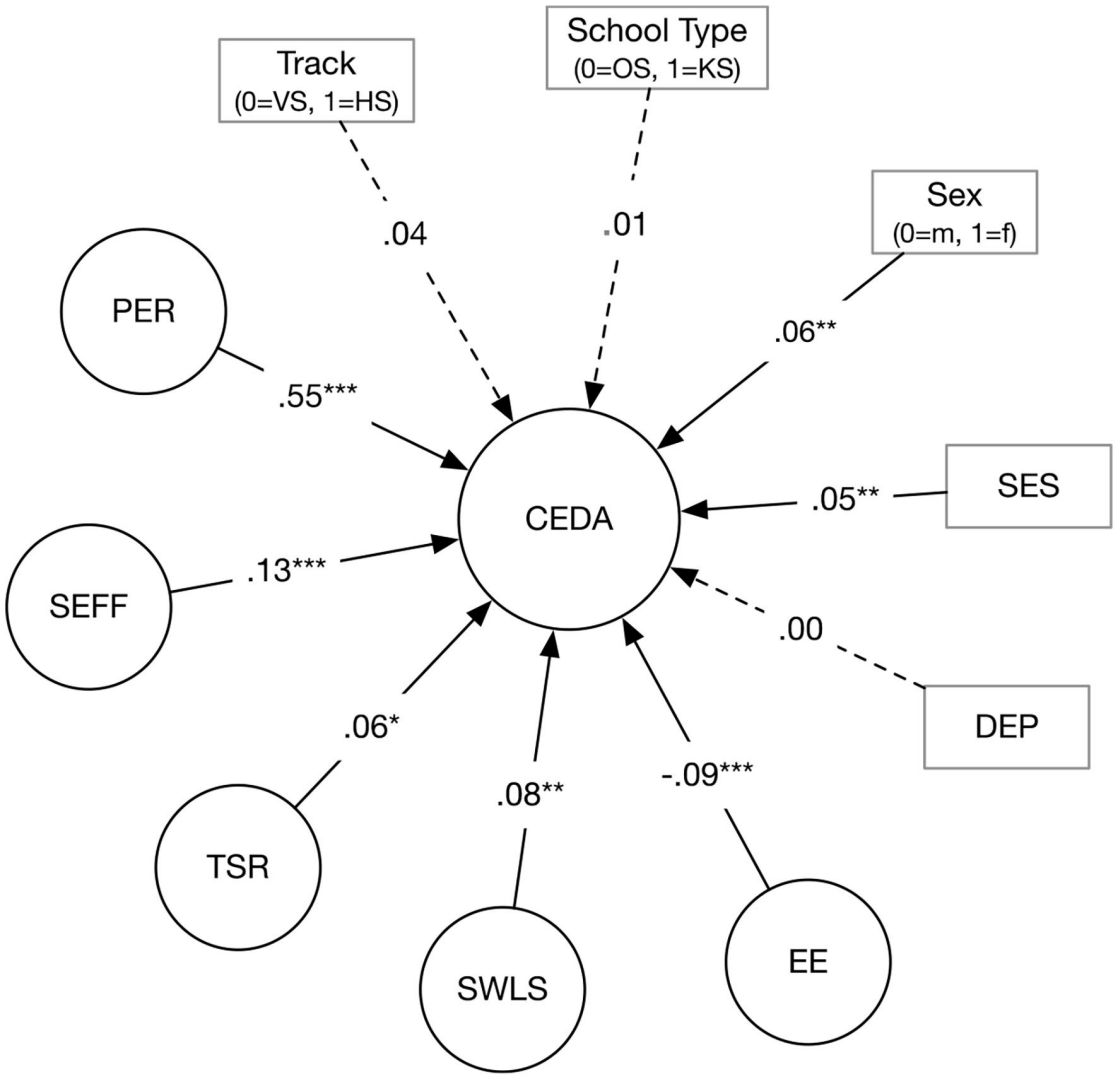
The relationship between schoolwork engagement (CEDA) and assumed correlates. VS, vocational school; HS, high school; OS, ordinary school; KS, key school; m, male; f, female; EE, emotional exhaustion; DEP, depressive symptoms; SWLS, satisfaction with life; TSR, teacher-student relationship; SEFF, self-efficacy; PER, perseverance of effort; Dotted line, not significant path. *p < 0.05. **p < 0.01. ***p < 0.001.

The results of the SEM [χ^2^ = 2579.316, *df* = 600, *p* < 0.001, CFI = 0.946, SRMR = 0.049, RMSEA = 0.041, 90% CI (0.040, 0.043); see Figure 2] showed that after controlling for gender, school tracks, and socioeconomic status, students' engagement was positively related to perseverance of effort, self-efficacy, teacher-student relationships, and life satisfaction (β between 0.06 and 0.55, *p* < 0.05), whereas engagement was negatively related to emotional exhaustion (β = −0.09, *p* < 0.001). There was no significant relationship between engagement and depression (β = 0.00, *p* = 0.89).

Students who reported higher values on socioeconomic status also reported higher values on engagement (β = 0.06, *p* < 0.01). Regarding the relationship between gender and engagement, females reported higher levels of engagement (β = 0.06, *p* < 0.01) than males. No differences in engagement regarding school track and school type were found.

In the SEM, the explained variance in engagement by all presumed factors was *R*^2^ = 0.55.

## Discussion

In the current study, we aimed to validate a Chinese questionnaire for the assessment of schoolwork engagement (CEDA). We adapted the Finnish Schoolwork Engagement Inventory (Salmela-Aro and Upadyaya, 2012). Data analyses were based on a large sample of Chinese general high school and vocational high school students. We tested the factorial structure of the CEDA and its measurement invariance across school tracks, school types, and gender. To evaluate its construct validity, we examined its relations to emotional exhaustion, students' resources, life satisfaction, and depressive symptoms.

In line with previous findings, the one-factor model and the three-factor model of the CEDA fitted the data similarly well. We finally choose the one-factor model as three dimensions of schoolwork engagement were highly correlated with each other. Schaufeli et al. (2006) found in their original study similar results and pointed out that the one-factor structure should be preferred to avoid multicollinearity. In the present study, we freed one residual correlation in the final CFA model based on the modification indices. From a statistical perspective, freeing residual correlations according to modification indices should be critically evaluated because this approach may lead to chance capitalization (i.e., drawing a biased conclusion in a particular direction by chance; MacCallum et al., 1992). To counteract it, cross validations in independent samples are necessary (MacCallum et al., 1992). In the Finnish EDA, two residual correlations were allowed. Also, in the long student version of the UWES (with 14 items), 4–6 residual correlations were allowed in the cross-national validation study on student engagement by Schaufeli et al. (2002a). The authors justified this approach with the argument that testing measurement invariance of the model in different subgroups could be considered as cross validations.

Regarding the measurement invariance, the CEDA is metrically invariant across school tracks. This allows comparisons of indicators between general high school and vocational high school students. The CEDA shows a scalar measurement invariance across school types and gender indicating possible comparisons of the intercept of the latent construct between ordinary school students and key school students as well as between boys and girls. Further, the internal consistency estimate of the overall scale of student engagement is high.

Prior research work has revealed the positive impact of students' resources on engagement. In line with previous findings (e.g., Salmela-Aro and Upadyaya, 2014; Tuominen-Soini and Salmela-Aro, 2014; Teuber et al., 2020a,b; Tuovinen et al., 2020), students' engagement was positively associated with positive psychological traits such as self-efficacy and perseverance of effort. Also, a positive teacher–student relationship was positively linked to engagement.

Against our assumptions, there was no relationship between schoolwork engagement and depression. However, previous research also showed that the association between schoolwork engagement and depression tended to disappear when burnout was taken into account (Salmela-Aro and Upadyaya, 2014). Relying on the assumptions of the Demands-Resources Models, both emotional exhaustion (as the central strain dimension of burnout) and engagement lead to academic and psychological outcomes. In school-related contexts, whereas emotional exhaustion in learning is a more important predictor of psychological outcomes such as well-being and depression, schoolwork engagement is a more important predictor of academic outcomes such as academic performance and school dropout. The results of several Finnish studies indicated that schoolwork engagement is negatively associated with the level of academic demands and competitiveness of the school (Salmela-Aro and Upadyaya, 2012). Although general high schools and key schools are more competitive than vocational high schools and ordinary schools in China, contrary to these Western findings and our hypothesis, engagement did not differ in school types or school tracks. Our findings suggest that vocational high school students and ordinary school students are not *per se* less engaged than general high school students and key school students after controlling for other variables (i.e., students' resources, gender, and socio-economic status). This may be due to the prevalent high academic demands in the Chinese school system (OECD, 2019). Through promoting students' resources, their engagement may be facilitated.

Regarding gender-specific differences in student engagement, our findings show that in Chinese upper secondary schools, girls are more likely to engage in academic work than boys. This supports earlier findings [e.g., Lei et al. (2018)].

Several limitations of this study should be noted. Firstly, this study had a cross-sectional design. It was not possible to draw casual conclusions. Secondly, although we were able to recruit general high school students from different school types and socioeconomic backgrounds in Shanghai, vocational high school students were from only one school in a relatively small Chinese city. In the future, general and vocational high school students from more regions (including different metropolitans, middle-size cities, and rural areas) should be addressed to test whether there are regional differences [e.g., Talhelm et al. (2014)] in student engagement and associated psychological constructs. Thirdly, socioeconomic status was measured by a single item (the number of books in the household) in this study. Due to the complexity of the mechanism of family socioeconomic status and its impact on child development (Bradley and Corwyn, 2002), we suggest including more indicators such as parental education and family income to represent socioeconomic status in future studies. Fourthly, this study relied on students' self-reports; common method variance may partly explain some of the results. It could be an advantage to obtain multiple measures of some constructs (e.g., engagement from the teachers' perspective). Further, because of the dynamics and the fluctuation in engagement (Bakker et al., 2014), the experience sampling method can be another good alternative. However, this method requires considerable time investment from participants, and the data quality depends largely on their willingness to comply with instructions (Hektner et al., 2007).

Overall, the Chinese version of the Schoolwork Engagement Inventory (CEDA) showed good psychometric properties. Hence, this inventory can be used for the assessment of student engagement in Chinese general high school and vocational high school contexts. Furthermore, it enables cross-cultural comparison studies that are associated with schoolwork engagement.

## Data Availability Statement

The raw data supporting the conclusions of this article will be made available by the authors, without undue reservation.

## Ethics Statement

The studies involving human participants were reviewed and approved by Bielefeld University. Written informed consent to participate in this study was provided by the participants' legal guardian/next of kin.

## Author Contributions

ZT conceptually designed the study, carried out analyses, interpreted the results, and drafted and revised the manuscript. XT contributed to the conceptual design of the study, guidance of analyses, and interpretation of the results and reviewed and revised drafts of the manuscript. KS-A reviewed and revised drafts of the manuscript. EW reviewed and revised drafts of the manuscript. All authors read and approved the final manuscript.

## Conflict of Interest

The authors declare that the research was conducted in the absence of any commercial or financial relationships that could be construed as a potential conflict of interest.
